# Probiotics: A Little Help for Enteral Nutritional Therapy in Critically Ill Adults

**DOI:** 10.3390/ijms26178458

**Published:** 2025-08-30

**Authors:** Graciele Magda de Almeida, Mariana Buranelo Egea

**Affiliations:** 1 Faculty of Agronomy, Federal University of Goiás, Goiânia 74690-900, Goiás, Brazil; gra.nutri@hotmail.com; 2 Goiano Federal Institute, Campus Rio Verde, Rio Verde 75901-970, Goiás, Brazil

**Keywords:** mechanical ventilation, intestinal microbiota, bioactive compounds, diarrhea, tube feeding

## Abstract

The administration of enteral nutritional therapy (ENT), combined with the use of probiotics, is considered a proactive therapeutic strategy that can modulate the intestinal microbiota, resulting in beneficial effects on intestinal integrity and function, as well as on the immune system of patients. This review aimed to find evidence on the clinical effects of probiotic administration in treating patients using ENT. An integrative search was performed to select scientific articles on the use of probiotics in ENT published in the last 10 years (2014–2025) using PubMed, ScienceDirect, Scielo, and Google Scholar databases. The descriptors used in the search were “probiotics” AND “enteral nutrition” OR “tube feeding” AND “adults” AND “critical illness”. Retrospective studies, pilot single/double-blind placebo-controlled clinical trials, and randomized trials investigating the effects of probiotic supplementation in enteral nutrition were included. A review of 21 manuscripts was conducted, in which all patients received ENT with probiotics, with 14 monitored in the ICU, 4 in the ward, and 3 at home. All 21 studies reviewed included a control group using enteral nutrition alone or a placebo, and some also included the study of other treatments. All studies demonstrated clinical benefits of some nature for patients who received enteral nutrition associated with the use of probiotics, such as reduced hospitalization time, improvement in the gastrointestinal tract, reduction in diarrhea associated with the use of antibiotics and inflammatory and immunological responses, and reduction in the incidence of pneumonia associated with mechanical ventilation. Probiotic supplementation in adult patients using enteral nutritional therapy demonstrates benefits that help promote health and improve intestinal microbiota composition. No side effects or adverse risks have been reported.

## 1. Introduction

**Figure 1:**
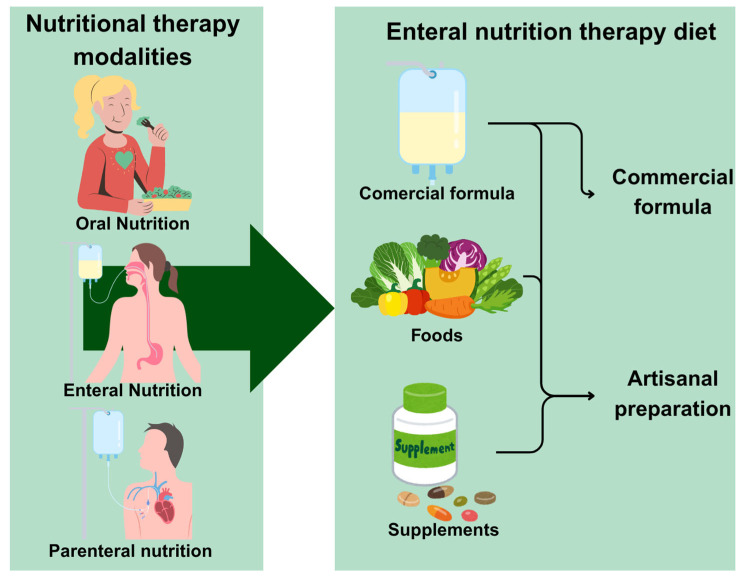
Nutritional therapy modalities (oral, enteral, and parenteral) and types of foods (commercial formula, foods, and supplements) can be used in the enteral nutritional therapy diet.

Nutritional therapy is defined as a set of therapeutic procedures aimed at maintaining or recovering the patient’s nutritional status, preventing and/or treatment of malnutrition, improvement of the immune and healing response, and modulating the response to clinical and surgical treatments, as well as prevention and treatment of infectious and non-infectious complications associated with the disease and the therapy used. This therapy can use oral, enteral, or parenteral routes (Figure 1). Once these goals are achieved, nutritional therapy improves quality of life, reduces the length of hospital stay, and decreases mortality [1].

Enteral Nutrition Therapy (ENT) is a fundamental strategy for nutritional support for critically ill patients. ENT is indicated exclusively or in addition to oral feeding, especially in malnourished patients or those at nutritional risk who have preserved or partially compromised absorptive capacity but whose oral route is insufficient to meet adequate nutritional demands [2].

With the popularization of ENT, it is now a form of nutrition that can be administered in the hospital and at home when the patient is discharged. Both in the hospital and at home, ENT can be administered with commercial formulas (usually in a closed system), blended foods (artisanal preparation), or a combination of both (Figure 1) [3].

ENT can also incorporate natural foods, industrialized formulas, or isolate nutrients and bioactive compounds as supplements (Figure 1). Among the bioactive compounds are probiotics, which are live microorganisms that, when administered in adequate quantities, confer health benefits to the host [4]. Scientific evidence has shown that, in appropriate doses, probiotics play a crucial role in modulating the immune system; preventing and treating gastrointestinal disorders such as diarrhea and inflammatory diseases; and reducing inflammatory processes and food intolerances [5,6]. Studies also indicate their action on the gut–brain axis, with the potential to reduce anxiety symptoms [7].

It is important to emphasize that ENT is an often-neglected topic. Therefore, it is tough to standardize efficient procedures regarding adequate patient nutrition that contribute to improving the patient’s overall condition. Finding alternatives that can contribute to patient improvement—such as using probiotics as an adjunct—is essential to inform healthcare professionals’ clinical decisions. Due to the above, the objective of this study was to review the literature published over the last decade on the adjuvant use of probiotic microorganisms in Enteral Nutrition Therapy.

The research was conducted in the SCIELO, ScienceDirect, SCOPUS, and Springer databases, focusing on studies published between 2014 and 2025. The descriptors used were: “probiotics” AND “enteral nutrition” OR “tube feeding” AND “adults” AND “critical illness”. The inclusion criteria included any clinical studies involving critically ill adults (≥18 years) who used ENT and a probiotic-based intervention. The review included all studies that used probiotic microorganisms (isolated or combined strains) in conjunction with ENT regardless of the patient’s underlying disease. Retrospective studies, pilot single/double-blind placebo-controlled clinical trials, and randomized trials investigating the effects of probiotic supplementation in enteral nutrition were included. The clinical outcomes analyzed included the incidence of diarrhea, immune function, length of hospital stay, mortality, and infections. Additionally, the review identifies gaps in the current literature and proposes directions for future research in this area.

## 2. Enteral Nutritional Therapy (ENT)

Enteral Nutrition Therapy (ENT) is a therapeutic procedure used for special purposes administered through a tube, alone, or in combination with oral nutrition. ENT may be indicated to reduce the energy deficit and insufficient dietary intake, thereby meeting the body’s energy demands, minimizing metabolic changes, and preventing the loss of lean mass—factors that contribute to malnutrition [8]. The ENT procedure can be performed in a hospital, an outpatient setting, or at home [2].

Nutritional and otorhinolaryngological assessments are initially considered to identify nutritional risk and support clinical decision-making [9]. In addition, it is essential to consider the patient’s clinical conditions, such as neurological diseases, neoplasms, trauma, and sepsis, among others, which often require enteral nutritional support [9].

**Figure 2:**
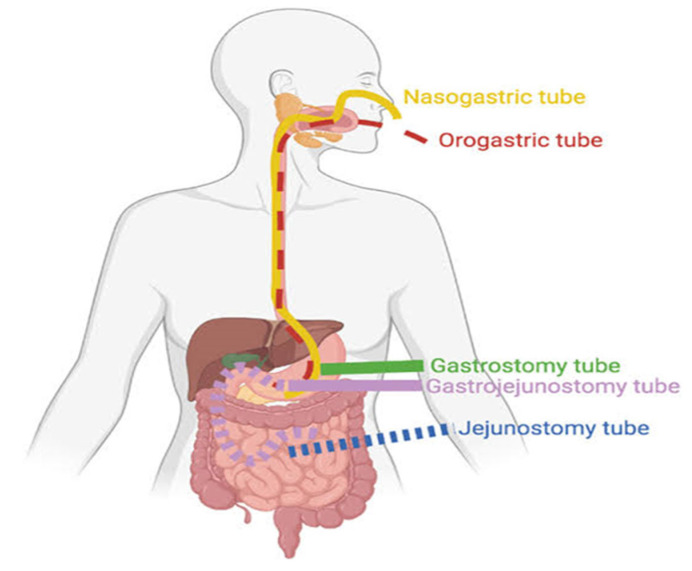
Positions of access through the nose or oral to stomach (nasogastric or orogastric tubes), stomach (gastrostomy tube), jejunum (jejunostomy tube), or jejunostomy tube for enteral nutritional therapy.

For ENT to be effective, the patient must also demonstrate viability of the gastrointestinal tract (GIT), verifying whether the digestive system has full or partial capacity for digestion, absorption, and metabolism of nutrients [10]. If the GIT is functional, ENT can be administered through silicone or polyurethane tubes, which are flexible and biocompatible materials available in various sizes and models. Depending on the need, these tubes can be positioned in the gastric or post-pyloric site. In specific cases, direct surgical access to the stomach (gastrostomy) or jejunum (jejunostomy) is used, as illustrated in Figure 2.

The choice of enteral access type should consider variables such as anatomical condition, current clinical status, risk of aspiration, and the estimated duration of therapy [11]. The nasogastric or nasoenteric route, through natural orifices, is recommended for short-term use (<4 weeks). In comparison, gastrostomy and jejunostomy are preferable in cases of prolonged need (more than 4 to 6 weeks), as they provide greater comfort and reduce complications such as sinus disease and pressure ulcers [12].

ENT administration can be administered continuously (with an infusion pump), intermittently (by gravity), or as a bolus (with a syringe). The choice of method depends on the clinical condition, gastrointestinal tolerance, and therapeutic goals. There is still no absolute consensus on the ideal method, and the decision is based on clinical evidence and individualized medical evaluation [13]. In clinically stable patients, ENT can be maintained at home for long periods with adequate monitoring [14,15]. In this scenario, Home Enteral Nutrition Therapy (HENT) is defined as nutritional support provided outside the hospital environment, with objectives that include humanizing care, reducing infections, shortening hospital stays, and saving health system resources [16]. Selection for HENT considers criteria such as clinical stability, patient and family acceptance, and caregiver training to ensure safe therapy performance [17].

To ensure the effectiveness and safety of HENT, continuous monitoring by a multidisciplinary team is essential. Nutritionists work on prescribing and adapting diets; doctors monitor clinical conditions; nurses train caregivers and oversee the preparation and administration of diets; speech therapists assess the risks of aspiration; and pharmacists contribute to the management of drug interactions with nutrients. At home, care is often assumed by family members or caregivers. To this end, it is essential to provide technical training and continuous support by the healthcare team, aiming to ensure patient safety and reduce the physical and emotional burdens on caregivers [18,19].

Specialized monitoring contributes not only to treatment adherence but also to preventing complications and promoting health, ensuring the patient’s quality of life [20].

## 3. Probiotics: Definition, Characteristics, and Potential Health Effects

Probiotics are defined as live microorganisms that, when administered in adequate amounts, provide health benefits to the host [4]. They primarily act in the gastrointestinal tract, promoting digestion, inhibiting pathogens, and stimulating the production of bioactive substances, with impacts on both intestinal and systemic health [21].

Gut dysbiosis is the imbalance in the proportion of microorganisms that comprise the healthy microbiota, characterized by the loss of commensal bacteria and the excessive growth of pathogenic microorganisms. In critically ill patients, gut dysbiosis has been associated with adverse outcomes, such as increased susceptibility to nosocomial infections, sepsis, and multiple-organ failure. In contrast, the symbiosis between the gut microbiota and the host can be optimized by pharmacological or nutritional interventions in the gut microbial ecosystem, such as the use of probiotics [22,23,24].

Regular consumption of probiotics has also been associated with preventing and treating conditions such as diarrhea and inflammatory bowel disease. It contributes to the prevention of chronic non-communicable diseases [25]. In addition, probiotics play a crucial role in modulating the immune system, optimizing nutrient absorption, and preventing cardiovascular and metabolic diseases [26].

Probiotics may reconstitute the microbiota and benefit patients by inhibiting pathogen growth or replacing pathogenic bacteria in the gastrointestinal tract, reducing bacterial translocation to the bloodstream and distant organs, preventing oropharyngeal colonization by pathogenic bacteria, and reducing the risk of microaspiration pneumonia [26,27,28].

Thus, probiotics emerge as a relevant therapeutic alternative for balancing the intestinal microbiota and modulating the immune system, in addition to exhibiting anti-inflammatory properties that can be explored in the treatment of various clinical conditions, including in critically ill patients [29,30].

Dysbiosis can occur in patients undergoing ENT procedures due to insufficient micronutrients and dietary fiber, essential fermentable substrates for producing short-chain fatty acids (SCFAs) by the intestinal microbiota. Additionally, the enteral route can eliminate the influence of oral bacteria that usually enter the digestive tract when food and saliva mix, which can contribute to changes in the intestinal microbiome composition [31,32]. Lower microbial diversity in these patients may be associated with the emergence of symptoms of ENT intolerance [33].

The gut microbiota also regulates the gut–brain axis, directly influencing neurological, immunological, and behavioral aspects [34]. In critically ill patients, there is evidence that dysbiosis can contribute to the development of delirium, cognitive alterations, and neurological dysfunctions. By modulating the gut microbiota, probiotics can benefit the central nervous system by producing neurotransmitters, neuroactive metabolites, and reducing systemic inflammatory cytokines, constituting a promising therapeutic approach in managing neuropsychiatric complications [35].

Approximately 30% of patients fed via an enteral tube have gastrointestinal complications such as vomiting, high gastric residual volume, diarrhea, constipation, and abdominal distension, which compromise the adequacy of nutritional supply and are associated with worse clinical outcomes [36,37]. In this sense, probiotic supplementation combined with ENT can reduce the rate of infectious and gastrointestinal complications (diarrhea, vomiting, gastric residual volume, constipation, and abdominal distension), improving clinical outcomes, such as shorter time on mechanical ventilation, stay in the intensive care unit, and mortality of critically ill patients [23,33,38].

In addition to probiotics, the emerging concepts of postbiotics and paraprobiotics stand out, which have been investigated as safe and effective alternatives for managing intestinal microbiota. Postbiotics refer to a preparation of inanimate microorganisms and/or their components that confer a health benefit on the host [39]. Thus, the literature suggests that bioactive compounds produced by microorganisms during fermentation, such as SCFAs, enzymes, and antimicrobial peptides, can be considered postbiotics [40]. Paraprobiotics consist of microorganisms inactivated (intact or broken) or crude cell extracts, which, when administered (orally or topically) in adequate amounts, confer a benefit to the human or animal consumer [41].

Although relatively recent and widely discussed, these approaches may offer advantages in clinical situations where live microorganisms (probiotics) pose a risk, as is the case with immunosuppressed patients. There are reports in the literature of cases of bacteremia, fungemia, endocarditis, liver abscess, pneumonia, and sepsis associated with the use of probiotics in individuals with profound immunosuppression or high-risk groups [42]. Individualized assessment and rigorous monitoring are crucial to ensure the safety of probiotic therapy in these cases. It is recommended that high-risk patients be excluded from standard supplementation protocols [43].

## 4. Probiotics as a Supplement in ENT: The Adult Experience

Critical illnesses, other severe pathologies, and medical interventions can lead to changes in the gastrointestinal tract, resulting in a loss or decrease in the intestinal microbiota and an overgrowth of potentially pathogenic bacteria. The administration of probiotic bacteria to patients in critical health conditions can restore the intestinal microbiota balance and yield positive results in immune and gastrointestinal function. Table 1 and Table 2 were organized by microorganism or combination of microorganisms to establish the benefits obtained for each case. Table 1 presents the study design and participant characteristics of all studies identified through the previously described search and reviewed here.

No reviewed work indicated side effects, bacteremia, or fungemia associated with using probiotic microorganisms in treatment. Therefore, probiotic microorganisms appear to be safe for use in the general population [4]. Furthermore, considering that all studies involved critically ill individuals (high-risk groups), especially for bacteremia and fungemia, these microorganisms appear to represent an alternative treatment without side effects for use in conjunction with ENT.

**Figure 3:**
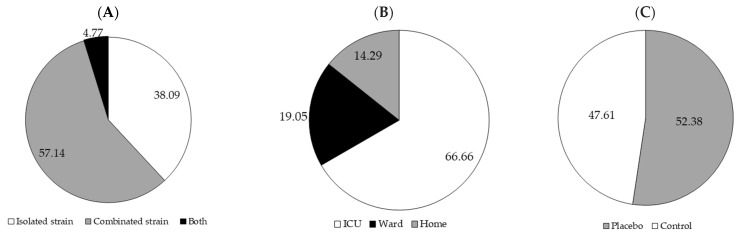
(**A**) Percentage of studies that used isolated or combined strains, (**B**) percentage of studies that were developed using the patient in enteral nutritional therapy in different locations, and (**C**) percentage of studies that used a control group or placebo.

The 21 studies analyzed included various population groups, mainly adult and elderly patients (mean age 63.4 years) in critical clinical conditions. Most participants were admitted to ICUs, often on mechanical ventilation, including patients with multiple traumas, severe traumatic brain injuries, senile sepsis, and postoperative conditions such as gastric cancer. Individuals on HENT with probiotics alone or in combination (Figure 3A), both with neurological disorders and geriatrics with multiple comorbidities, were also included. In some studies, patients were admitted to respiratory care units or medical wards with support from ENT specialists. The manuscripts presented robust methodological designs, such as randomized, double-blind clinical trials and prospective studies, ensuring greater scientific rigor in comparing the intervention and control groups.

All patients in the studies evaluated in this review received enteral nutritional therapy; 14 were monitored within the ICU (66.66%) [44,45,46,47,50,52,53,55,56,59,60,61,62,63], 4 were monitored in the wards [51,54,57,58], and 3 were monitored at home [18,48,49,64] (Figure 3B). ENT, whether in a hospital or home environment, has advantages and disadvantages that must be carefully assessed based on the patient’s clinical condition.

In a hospital environment, the primary advantage is constant monitoring by a multidisciplinary team, enabling immediate adjustments to nutritional prescriptions and the management of complications, such as intolerances or infections. However, prolonged hospital stays can increase the risk of nosocomial infections, cause emotional distress, and lead to higher hospital costs [65].

Home ENT, on the other hand, offers the primary benefit of an improved quality of life, with greater comfort and enhanced family interaction, and reduces hospital costs. However, its main disadvantage is the need for caregiver training and the possibility of complications due to inadequate handling, such as tube obstruction or diet contamination [3]. Therefore, the choice between hospital and home therapy should consider both the clinical condition and the support available at home.

All 21 manuscripts included a control group (52.38%) [50,51,53,54,56,57,58,62,63,64] using enteral nutrition alone or a placebo [18,44,45,46,47,48,52,55,59,60,61,63] (Figure 3C). Some of them included the study of other treatments [18,53,57,58]. The placebo group, in most cases, received an inert compound as a sachet with powdered glucose polymers or a capsule with microcrystalline cellulose, sterile cornstarch, potato starch, or magnesium stearate with an appearance identical to the probiotic treatment, allowing the psychological effect (placebo effect) to be controlled, favoring the blinding of the experiment and reducing bias. The control group, which uses only ENT, is a basis for comparison, as it is more common and ethically acceptable. However, it does not eliminate the placebo effect, which can compromise interpreting the results in studies with human participants [66].

Critically ill patients in the ICU, especially those on mechanical ventilation and receiving ENT, often present with significant gastrointestinal dysfunctions, such as abdominal distension, constipation, malabsorption, and, mainly, recurrent diarrhea. These symptoms can be aggravated by several factors common in the ICU setting, such as prolonged use of broad-spectrum antibiotics, sedatives, the systemic inflammatory response itself, and ENT, which, although essential, can impact the integrity of the intestinal mucosa and promote significant changes in the gastrointestinal microbiota. The interaction between these elements contributes to the impairment of the intestinal barrier function, increasing the risk of infections and worsening the clinical prognosis [67,68].

**Figure 4:**
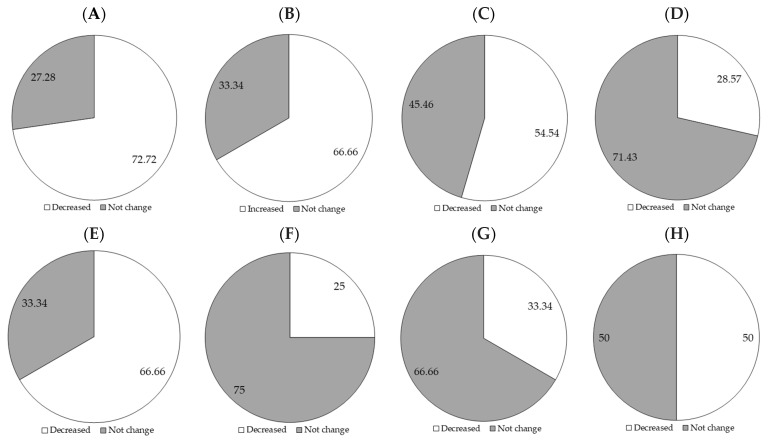
Percentage of occurrence of clinical outcomes, including the incidence of diarrhea (*n* = 11) (**A**), immune function (*n* = 6) (**B**), length of hospital stays (*n* = 11) (**C**), mortality (*n* = 7) (**D**), infection occurrence (*n* = 12) (**E**), mechanical ventilation use (*n* = 4) (**F**), frequency of vomiting (*n* = 3) (**G**), and ventilator-associated pneumonia (*n* = 4) (**H**).

Figure 4 shows the outcomes found when probiotic microorganisms are used in ENT in adult patients with critical illness. This relative percentage was calculated based on studies controlling for the specific outcome. In general, it was possible to see that a higher percentage of studies demonstrated a decrease in the incidence of diarrhea (72.72%) and infection occurrence (66.66%), while a higher percentage demonstrated that there was no change in the length of hospital stays (54.54%), mortality (71.43%), mechanical ventilation (75%), weight loss (100%), and frequency of vomiting (66.66%). Meanwhile, studies that evaluated immune function showed an increased prevalence (66.66%).

**Figure 5:**
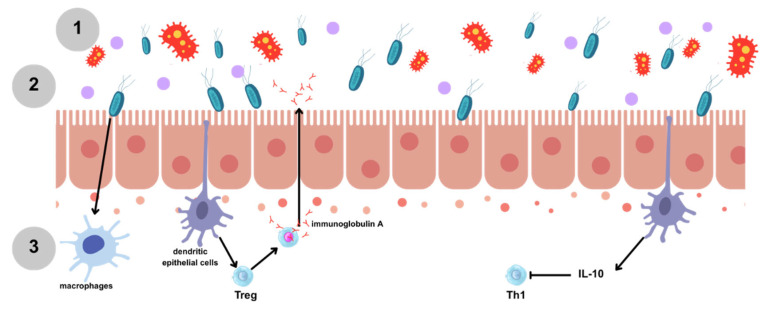
Possible mechanisms of action of probiotic use in critically ill patients are (**1**) antimicrobial activity, (**2**) physical protection and alteration of the intestinal lumen, and (**3**) immunomodulating and anti-inflammatory activities. Treg: Regulatory T cells; Th1: T helper 1 cells; and IL-10: Interleukin 10.

In this context, the use of probiotics in ICU patients appears to be a promising therapeutic approach because it contributes to restoring the balance of the intestinal microbiota, improving gastrointestinal symptoms through several mechanisms: (1) inhibition of colonization by pathogens and production of antimicrobial substances (Figure 5(1)); (2) strengthening of the intestinal epithelial barrier, decreased intestinal pH, improvement in intestinal motility, and absorption of nutrients (Figure 5(2)); and (3) alteration in cytokine production in macrophages and dendritic cells (such as IL-10), modulating of the inflammatory response (Figure 5(3)).

Therefore, the use of probiotics as adjuvant therapy in critically ill patients in the ICU can contribute to maintaining gastrointestinal function, preventing secondary infections, and improving immunological status, thereby favoring clinical recovery and reducing the length of hospital stay [69,70,71].

Among the reviewed studies (Table 2), isolated strains and genera were used (use of a single species/microorganism) [44,45,46,48,49,50,51,52,53], combined genera or strains (mixture of two or more) [18,47,54,55,56,57,59,60,61,62,63,64], or both [58] (Figure 3A). Both approaches have advantages and disadvantages that should be considered in light of the therapeutic objectives and the patient’s profile.

The main advantage of using isolated strains (as in studies [44,45,46,48,49,50,51,52,53,58]) is the ability to more clearly assess the specific effects of a single strain, which facilitates standardization, the study of mechanisms of action, and the reproducibility of clinical results. In addition, it is easier to ensure the stability, viability, and safety of the product [72]. However, its disadvantage is the limitation of its spectrum of action, as different strains exhibit varying properties, such as immune modulation, production of short-chain fatty acids, or competitive exclusion of pathogens [73].

Probiotics with combined strains (as in studies [18,47,54,55,56,57,58,59,60,61,62,63,64]) can offer synergistic effects, promoting a more comprehensive action on the intestinal microbiota, the inflammatory response, and the intestinal barrier function. This is especially advantageous in multifactorial conditions, such as inflammatory bowel disease, irritable bowel syndrome, or during antibiotic use. However, the main disadvantage is the complexity of evaluating individual effects, making it difficult to scientifically prove efficacy and increasing the risk of negative interactions between strains, where probiotic species inhibit each other. Additionally, there are possible challenges in the stability and formulation of the final product [74]. Furthermore, most studies do not include comparisons with treatments with isolated strains, demonstrating that it is still unclear whether this is due to synergistic interactions between strains or to the higher dose of probiotics [73].

Species of the genus *Limosilactobacillus* (former *Lactobacillus*) are Gram-positive bacteria that ferment carbohydrates, producing lactic acid, which creates an environment unfavorable to the growth of pathogens (Figure 5(1)). Species such as *Lactobacillus acidophilus*, *Lacticaseibacillus rhamnosus* (former *Lactobacillus rhamnosus*), and *Lacticaseibacillus casei* (former *Lactobacillus casei*) are effective in preventing and treating intestinal disorders, such as antibiotic-associated diarrhea and lactose intolerance. The lactic acid produced by these species helps to reduce intestinal pH, inhibiting the growth of pathogenic microorganisms, such as *Clostridium difficile* and *Salmonella* [75].

*L. rhamnosus* for 60 days [44,45] demonstrated the safety and feasibility of supplementation, preventing ventilator-associated pneumonia and other infections in critically ill patients, delaying the initiation of antimicrobial drugs, and decreasing the duration of this therapy. Selected strains of the species *L. rhamnosus*, identified as potential probiotic strains, are widely used in food formulations due to their high resistance to technological processes. Widely used in food formulations, *L. rhamnosus* contributes to intestinal homeostasis by secreting bioactive molecules such as proteins, polysaccharides, and lipoteichoic acids, which strengthen the intestinal barrier, attenuate oxidative stress, and modulate inflammatory responses [76].

*L. rhamnosus* stimulates IgA secretion and adheres to the intestinal mucosa via pili, enhancing mucosal defense and exerting long-lasting epigenetic effects [76,77]. Pilar structures on its surface facilitate adhesion to the intestinal mucosa, promoting a closer interaction with host cells. The extracellular vesicles released by the strain also carry these bioactive molecules and replicate many of the immunomodulatory and protective effects observed. Together, these mechanisms significantly contribute to the integrity of the intestinal mucosa, the regulation of the immune system, and the development of immunological tolerance, thereby reinforcing the role of *L. rhamnosus* as a crucial ally in promoting intestinal health [78,79].

*Lactiplantibacillus plantarum* (formerly *Lactobacillus plantarum*) is one of the most relevant species of lactobacilli and has been widely used as a probiotic due to its remarkable functional properties. Studies have demonstrated its efficacy in reducing the occurrence of diarrhea and improving tolerance to ENT after 60 days [46] and 12 weeks of use [48,49]. The high tolerance of *L. plantarum* to acidic pH and bile is due to mechanisms such as lipopolysaccharide production, regulation of amino acid metabolism, and increased proton pump activity [80,81,82].

In addition, *L. plantarum* exhibits antimicrobial activity against several pathogens, including *Listeria monocytogenes*, *Yersinia enterocolitica*, *Enterobacter cloacae*, *Enterococcus faecalis*, and *Escherichia coli* [83], which can be found in artisanal preparations [3]. This action occurs through the production of bioactive compounds, including organic acids (lactic, glycolic, and citric), fatty acids (stearic, palmitic, and octanoic), glycerides (monopalmitin and glycerol monostearate), and amino acids such as aspartic acid, glycine, and serine, which contribute to its bacteriostatic and bactericidal effects [84]. *L. plantarum* modulates the immune response, reducing inflammatory cytokines and stimulating the production of the anti-inflammatory IL-10 [85,86].

*L. casei*, commonly present in fermented foods, helps prevent antibiotic-associated diarrhea and *C. difficile* infections in administration periods of 7 [50] and 28 days [51]. Furthermore, studies indicate that its use can reduce the incidence of ventilator-associated pneumonia (VAP) (50%, Figure 4H) and colonization by resistant bacteria in the oropharyngeal cavity. However, it has not shown significant effects on mortality or length of hospital stay.

The beneficial effects of *L. casei* are attributed to several molecular mechanisms. The strain acts to modulate the intestinal microbiota through the production of organic acids, such as lactic acid, which inhibit the growth of pathogens and contribute to maintaining an acidic intestinal pH. It also exerts an anti-inflammatory action, reducing the activity of enzymes such as myeloperoxidase (MPO) and nitric oxide levels, which are associated with intestinal inflammatory processes. These mechanisms explain its role in preventing infections and modulating the immune system, with potential repercussions on mental health [87,88].

*Clostridium butyricum* [52] has been utilized for its beneficial effects on growth performance, immunity, and intestinal microbiota balance, particularly in situations of stress or inflammatory challenge [83]. Wang, Ke, Liu, and Qu [52] evaluated the exogenous administration of this strain on clinical outcomes in critically ill ICU patients. Although probiotic intake did not significantly improve primary clinical outcomes such as mortality and length of hospital stay, in critically ill patients, a reduction in fever time (percentage of hospital stay) and the incidence of constipation was demonstrated, indicating improvements in gastrointestinal recovery. Additionally, there was a trend toward a reduction in the load of transgenic bacteria in the intestine.

*Bacillus cereus* A05 improves immunomodulatory mechanisms in malnourished patients [53]. The study suggested that this occurs through the interaction of *B. cereus* with the gut-associated lymphoid tissue (GALT), resulting in the modulation of the immune system by B lymphocytes, increasing the intestinal response in malnourished patients. Since malnutrition leads to compromised intestinal barrier function, the intake of this probiotic may be more effective in these patients, resulting in a faster resolution of diarrhea.

Probiotic *Bacillus* species such as *B. cereus*, *B. clausii*, and *B. subtilis* exhibit high heat, gastric pH, and humidity stability, making them suitable for varied clinical and industrial uses. These strains contain antimicrobial substances, including bacteriocin, SCFAs, and organic acids, and can modulate gastrointestinal disorders through their antimicrobial and antiadhesive effects against pathogenic strains [89]. Additionally, they possess anticancer, antioxidant, and vitamin-producing properties. *Bacillus* probiotic strains have the merit of stability and their benefits as probiotics; however, they may present risks regarding the production of enterotoxins, the transfer of antibiotic resistance genes to other strains, cytotoxicity against normal cells, and the production of biogenic amines. Specifically, *B. cereus* poses a risk due to its ability to produce enterotoxin as a human pathogen [90,91].

The use of species of the genus *Bifidobacterium* for 14 days is associated with ENT [54]. This resulted in several benefits in treating gastric cancer patients undergoing chemotherapy, including improved regulation of the intestinal microbiota balance, improved nutritional status, improved levels of endotoxin and D-lactic acid, improved immunity, and reduced gastrointestinal symptoms.

The *Bifidobacterium* genus acts synergistically in the intestinal microbiota, competing with pathogens, modulating immunity, and producing SCFAs (short-chain fatty acids) such as acetate, propionate, and other antimicrobial metabolites. These effects result in reduced bacterial translocation, improved intestinal barrier function, and metabolic benefits [92,93,94].

Species belonging to the *Limosilactobacillus* and *Bifidobacterium* genera were used for 7 days [55,56,57] or throughout diarrhea [58], which resulted in a reduction in serum markers of inflammation such as C-reactive protein (CRP), Interleukin-6 (IL-6), and Tumor Necrosis Factor (TNF), and a lower incidence of gastrointestinal complications such as diarrhea, intestinal inflammation, dysbiosis, abdominal pain, and vomiting, in addition to favoring a faster return of intestinal function. The *Limosilactobacillus* and *Bifidobacterium* genera combination favors fiber fermentation, SCFA production, and immune modulation, reducing inflammation and strengthening the intestinal barrier [94,95,96,97].

*Limosilactobacillus* strains acidify the gut through the production of lactic acid and release bacteriocins, which inhibit pathogens such as *C. difficile*, thereby contributing to a balanced microbial environment. In addition, they favor the fermentation of fibers and the production of SCFAs essential for intestinal health. Their presence enhances microbial diversity, thereby strengthening intestinal homeostasis, which explains their use in therapies for dysbiosis, diarrhea, and inflammatory bowel disorders [98,99].

Furthermore, Zhao, Wang, Huang, Cui, Xia, Rao, Zhou, and Wu [57] combining fiber with these probiotic genera can reduce diarrhea, improve intestinal transit, and decrease gastrointestinal disorders in postoperative patients with gastric cancer on enteral nutrition. Fibers include fermentable prebiotics, which lead to specific changes in the composition and/or activity of the intestinal microbiota, benefiting the well-being and health of the host. For example, prebiotics that include fructo-oligosaccharides (FOS) and inulin have been shown in human studies to increase bifidobacteria concentrations. Bacterial fermentation of ingested fiber in the colon produces SCFAs, mainly propionic and butyric acids. These SCFAs offer several health benefits to the host, including providing fuel to colonocytes, regulating epithelial cell proliferation and differentiation, increasing colonic blood flow, reducing colonic pH, stimulating pancreatic secretions, promoting sodium and water absorption, and possibly influencing intestinal motility [100,101,102].

Although synergism is often discussed between strains of different genera, other studies have combined the action of other, less-studied genera. The combination of species from the *Limosilactobacillus*, *Bifidobacterium*, and *Streptococcus* genera, used for 14 days [47,59] in patients, demonstrated a lower incidence of pneumonia associated with mechanical ventilation and a shorter duration of ICU stay, as well as increased volume of enteral feeding and energy and protein intake during the first days of ICU admission.

Streptococcus are also homofermentative lactic acid bacteria with all Lactobacilli characteristics and are characteristic inhabitants of the intestinal tract. Like other lactic acid-producing bacteria, they balance the intestinal flora by acidifying the intestinal lumen, exerting a bactericidal and bacteriostatic effect that eliminates pathogenic bacteria sensitive to decreased pH. They also release enzymes that exert synergistic effects on digestion, alleviating symptoms of intestinal malabsorption, promoting adhesion with epithelial cells to form a functional barrier, minimizing secretion and inflammation resulting from bacterial infections, intensifying host cell signaling to reduce the inflammatory response, and reducing the production of inflammatory substances. Additionally, they have been observed to possess antioxidant capacity. *Streptococcus thermophilus*, in particular, is a strain that exhibits mechanisms such as the elimination of reactive oxygen species, metal ion chelation, and a chelating capacity [103,104,105].

The combination of species from the *Limosilactobacillus*, *Bifidobacterium*, and *Saccharomyces* genera was administered for 15 days [60,61]. It demonstrated a positive effect on the incidence of ventilator-associated pneumonia and sepsis and ICU and hospital stays in patients undergoing prolonged mechanical ventilation. Tzikos, Tsalkatidou, Stavrou, Thoma, Chorti, Tsilika, Michalopoulos, Papavramidis, Giamarellos-Bourboulis, and Kotzampassi [60] also demonstrated that this combination positively affected the incidence of surgical site infections in patients with severe polytrauma, significantly reducing overall infections and sepsis.

*Saccharomyces boulardii* and *S. cerevisiae* are antibiotic-resistant yeasts capable of surviving digestive conditions, making them valuable probiotics for managing diarrhea and enhancing mucosal immunity. Among their characteristics are the stimulation of the immune and non-immune mechanisms of the mucosa through antagonism/competition with potential pathogens, the activation of local macrophages and the modulation of IgA production, promoting changes in the profiles of pro- and anti-inflammatory cytokines and/or modulation of the response to food antigens, stimulation of mucin production and action on the tight junctions between epithelial cells, preventing the translocation of pathogens. They act in the regulation of gastrointestinal transit, in addition to improving the absorption of ions by the epithelial cells of the intestine, and improving different types of diarrhea (antibiotic-associated diarrhea, acute diarrhea, traveler’s diarrhea caused by bacteria, viruses, or parasites, and diarrhea related to enteral nutrition) [106,107,108].

The combination of *Limosilactobacillus*, *Bifidobacterium*, and *Enterococcus* genera administered for 15 days [62,63] has been shown to reduce serum levels of inflammatory factors (IL-6, TNF-α, and CRP). The duration of hospitalization and rates of pulmonary infection were also significantly reduced. Post-treatment serum albumin and prealbumin levels, and statistically lower intestinal fatty acid binding protein, diamine oxidase, D-lactate, and 28-day mortality were also evident. The combined enteral nutrition regimen with probiotics can significantly improve intestinal function, nutritional status, and prognosis of patients [63]. Wan, Wang, Zhang, Zhang, Lu, Li, and Yi [62] reported that after the administration of probiotics, the levels of inflammatory factors such as endothelin-1 (ET-1), CRP, and TNF decreased significantly, and there was also a reduction in hospital stay and pulmonary infection rates in patients with severe traumatic brain injury.

Enterococcus strains belong to the lactic acid bacteria (LAB) and are reported to produce antimicrobial compounds, including bacteriocins. Bacteriocin production has been applied to preserving a wide range of food products and is considered a probiotic trait. Enterococcal bacteriocins are recognized for their broad antimicrobial activity, including foodborne Gram-positive pathogens and Gram-negative bacteria. In addition, some bacteriocins possess antifungal and/or antiviral activity and can also inhibit sporulating bacteria, such as *C. botulinum* and *B. cereus*; in some cases, they can even inhibit endospores. These characteristics provide the rationale for identifying bacteriocinogenic Enterococcus strains as essential candidates for applications in food, human health, and controlling foodborne pathogens. Bacteriocin-producing probiotics may compete with intestinal pathogens for colonization or modulate microbiota homeostasis, thereby reducing gastrointestinal disease or complications [109,110]. Although some strains are safe and well-studied, the *Enterococcus* genus includes potentially pathogenic and antibiotic-resistant strains, especially in hospitals. Therefore, the clinical use of Enterococcus probiotic strains should be restricted to well-characterized strains that are free from virulence and antibiotic resistance genes, with rigorous safety testing [111].

The combination of *Limosilactobacillus*, *Bifidobacterium*, and *Lactococcus* genera used for 4 months improved constipation and stool consistency in these patients [64]. These probiotic strains demonstrate the ability to strengthen the intestinal barrier function after immunologically induced stress, in addition to stimulating IL-10 levels [112]. Interleukins are polypeptides (proteins) produced by leukocytes (mainly T lymphocytes, macrophages, and eosinophils) in response to microorganisms and other antigens, which mediate and regulate immunological and inflammatory reactions. IL-10 is one of the most important anti-inflammatory cytokines in the immune response and is an essential immunomodulator. It is produced by activated T cells (Th2), macrophages and monocytes, B cells, and mast cells (Figure 3). IL-10 has attracted interest due to its role as a key modulator of the innate immune response in inflammatory processes, as it controls the immune system after invasion by opportunistic microorganisms [113].

*Lactococcus lactis* is a nonpathogenic lactic acid bacterium that can ferment lactose. Lactose fermentation by *L. lactis* produces acetate that reduces the intracellular pH of some pathogenic bacteria, such as *Salmonella*, *Pseudomonas*, *Vibrio*, and *Leptospira* strains, impairing their motility and slowing the rotation of their flagella. These effects highlight the potential use of *L. lactis* in preventing infections caused by multiple bacterial species. *L. lactis* strains, including genetically modified ones, induce the production of anti-inflammatory cytokines, such as IL-10, which promotes the regulation of dendritic cells and an increase in regulatory T cells, thereby contributing to the modulation of intestinal immunity. Thus, *L. lactis* acts as a probiotic through producing antimicrobial compounds, immune modulation, and reinforcement of the intestinal barrier [114,115].

The combination of *Limosilactobacillus*, *Bifidobacterium*, and *Lentilactobacillus* genera for 60 days [18] was shown to be safe and easy to administer. Still, it did not alter the incidence of infections or modulation of inflammation in elderly individuals treated with enteral nutrition.

*Lentilactobacillus* is a heterofermentative bacterium that produces lactic acid and acetic acid during fermentation. The *Lentilactobacillus* genus, particularly the species *Lentilactobacillus kefiri* and *L. buchneri*, has emerged as a promising probiotic with several beneficial effects on human health. These strains have demonstrated antimicrobial activity against pathogens and their toxins, exhibit immunomodulatory effects, and induce beneficial metabolic effects. Notably, the strain *L. kefiri* CIDCA 8348 has been shown to increase the expression of anti-inflammatory interleukins, such as IL-10, and reduce pro-inflammatory cytokines, including IFN-γ and IL-1β, thereby contributing to the relief of intestinal inflammation and strengthening the intestinal epithelial barrier. Another important mechanism of *L. kefiri* is its direct antimicrobial action, through the production of bacteriocins that inhibit pathogens such as *Salmonella*, *Escherichia coli*, and *Clostridioides difficile*, in addition to competing with these microorganisms for adhesion to intestinal epithelial cells [116,117].

However, it seems clear that it is worthwhile to focus future studies on strains of the *Lacticaseibacillus* genus and the combination of these strains with *Bifidobacterium*. These are two genera that have been widely described in the literature for their role in the intestinal microbiota and are part of several probiotic supplements that can be easily found on the market. It is worth noting that the use of probiotics is not without risks, especially for ENT patients who have access to feeding through a tube, since cases of bacteremia, fungemia, endocarditis, liver abscess, pneumonia, and sepsis associated with the use of probiotics have been reported in a state of profound immunosuppression or high-risk groups [42].

This review systematizes studies on probiotics in conjunction with enteral nutrition in critically ill patients. The lack of standardization regarding strain, dose, and time of use limited the ability to draw robust conclusions, requiring additional controlled studies. Other authors had already reported this limitation regarding the review of the use of probiotics in clinical treatments [118]. It is also necessary to mention that the studies cited in this review demonstrated heterogeneity of the diseases that affected the patients, since many of them only used the fact that the patient was in a critical condition in the ICU, sex, and age as selection criteria. Furthermore, due to the specificity of the enteral feeding route, many studies had reduced sample sizes.

Another limitation of this review is that many studies vaguely include the type of supplement used, often describing only the genus of the probiotic without describing the strain or the viable quantity added. This makes it impossible to replicate these studies and hinders the scientific method in general.

Furthermore, due to the different natures of the studies, no formal assessment of risk of bias was performed. The absence of these procedures may compromise the robustness and increase uncertainty about the validity of the studies, even if some clinical statements can be made based on the narrative review carried out here.

## 5. Conclusions and Future Perspectives

This review is essential for assessing the effect of probiotic treatment on enteral nutrition in various clinical settings. The mechanisms by which probiotics act still need to be better elucidated, and more research is needed to understand which species and/or dose has the most significant impact on treating these patients.

Although the use of probiotics in enteral nutrition therapy has gained increasing attention due to their ability to modulate the intestinal microbiota, improve epithelial barrier function, and favorably influence the host immune response in some clinical contexts, future perspectives point to a more specific, personalized, and safe use, as new technologies and evidence are consolidated, including the use of specific strains, improved delivery technologies and integration with individual microbiota data, with the potential to positively impact several clinical settings.

To this end, more high-quality clinical trials, standardization of strains and formulations, and clear safety protocols are needed. Priority should be given to strain combinations, since different strains may exert complementary or synergistic effects, and the identification of the most effective doses, duration, and frequency of administration. Thus, these approaches would provide more solid evidence for clinical application and guide personalized nutritional or therapeutic strategies.

## Figures and Tables

**Table 1:** Study design and participant characteristics of revised manuscripts on the adjuvant use of probiotic microorganisms in Enteral Nutrition Therapy (ENT) published over the last decade (2014–2025).

Reference	Study Design	Studied Group	Study Duration	No PTP	No Male PTP	No Female PTP	Age (INT)	Age (PLA or CON)
[44]	Prospective, randomized, double-blind, controlled clinical trial	ICU patients on mechanical ventilation ≥ 72 h	stay in the ICU or until 60 d	150	88	62	58.8 ± 17.0	61.2 ± 15.4
[45]	Prospective, randomized, double-blind clinical trial	ICU patients on mechanical ventilation ≥ 72 h	60 d	2.650	1.587	1.063	60.1 ± 16.2	59.6 ± 16.8
[46]	Randomized double-blind controlled clinical trial	Patients with >48 h of ICU admission	60 d	208	134	74	62.1 ± 15.7	62.6 ± 14.5
[47]	Randomized controlled trial	Critically ill patients undergoing mechanical ventilation for >48 h in the ICU	14 d	102	55	45	59.1 ± 12.9	57.5 ± 14.5
[48,49]	Randomized, double-blind, and placebo-controlled	Cancer patients receiving home enteral nutrition	12 w	35	27	8	60.0 ± 10.9	61.1 ± 8.9
[50]	Parallel group randomized trial	Patients hospitalized in the ICU	7 d	32	25	7	59.9 ± 15.6	57.5 ± 15.0
[51]	Prospective, randomized, controlled study	Patients on mechanical ventilation from medical wards	28 d	150	62	88	68.9 ± 18.4	73.1 ± 13.2
[52]	A single-blind, randomized controlled trial	Patients admitted to the respiratory intensive care unit (RICU)	until death or discharge, and evaluated after 15 days	61	34	27	81 (61–95)	81 (70–96)
[53]	Randomized, prospective, double-blind study	Patients hospitalized in the ICU	5 d	58	ND	ND	65.0 ± 20.7	70.8 ± 18.1
[54]	Retrospective study	Patients with gastric cancer undergoing chemotherapy	14 d	80	43	37	58.3 ± 8.3	57.6 ± 8.3
[55]	Randomized, double-blind study	Critically ill patients on mechanical ventilation in the ICU	7 d	60	40	20	60	55
[56]	Cross-sectional study	Patients with COVID-19 who were admitted to the ICU tolerated approximately 80% of ENT	7 d	100	ND	ND	45.63	ND
[57]	Prospective randomized-controlled trial	Postoperative patients with gastric cancer (stage II or III tumors)	7 d	120	62	58	66.5 ± 7.1	63.5 ± 8.5
[58]	Retrospective, analytical, and longitudinal study	Patients admitted to the hospital	While the diarrhea lasted	75	32	43	71.8 ± 7.9	
[59]	Randomized controlled triple-blind clinical trial	Patients hospitalized in the ICU	14 d	38	23	15	38.5 ± 17.94	47.61 ± 22.51
[60]	Multi-center, randomized, double-blind, placebo-controlled trial	Surgical site infections in multiple-trauma patients on mechanical ventilation in the ICU	15 d	103	90	13	38.4 ± 16.9	44.1 ± 13.9
[61]	Randomized, controlled study	Multi-trauma patients on mechanical ventilation in the ICU	15 d	112	94	18	38.1 ± 17.2	43.8 ± 14.4
[62]	Prospective study	Patients with severe traumatic brain injury are being transferred from the hospital	15 d	76	36	40	35.9 ± 13.1	38.6 ± 11.3
[63]	Retrospective study	Patients with senile sepsis	2 w	108	61	47	71.3 ± 7.7	
[64]	Randomized open-label intervention study	Patients on home enteral nutrition with neurological disorders	4 m	20	ND	ND	75.2 ± 4.3	
[18]	Pilot, Double-Blind, Placebo-Controlled Study	Geriatric patients on home enteral nutrition with different comorbidities	60 d	32	8	24	80.0 ± 10.2	79.3 ± 10.0

CON: control group; d: days; ICU: Intensive care unit; INT: intervention group; m: months; ND: not described; PLA: placebo group; PTP: participants; w: weeks.

**Table 2:** Effects in adult patients of the adjuvant use of probiotic microorganisms in Enteral Nutrition Therapy (ENT) of revised manuscripts published over the last decade (2014–2025).

References	Main Results	Intervention
[44]	No comparison between groups was needed to analyze feasibility outcomes. The rate of VAP was 19%; bloodstream infection (19.3%); urinary tract infections (12.7%); skin and soft tissue infections (4.0%); and Bristol stool type 6 or 7 occurred in 133 (88.7%) of patients. The median stay in the ICU was 12 days, and in the hospital, it was 26 days.	1 capsule of Culturelle^®^ content 1 × 10^10^ CFU of *Lacticaseibacillus rhamnosus* GG (former *Lactobacillus rhamnosus*) was administered twice/day until discharge or death vs. placebo during the patient’s stay in the ICU
[45]	= diarrhea, antimicrobial use, length of hospital stay, and VAP.	1 × 10^10^ UFC de *L. rhamnosus* GG (i-Health Inc^®^)
[46]	= Days alive and out of the hospital to Day 60; nosocomial infection; mortality in the ICU and hospital; and overall quality of life at day 60, as assessed by median EQ-5D-5L VAS scores. No participant had more than one nosocomial infection.	1 capsule containing 2 × 10^10^ CFU of *L. plantarum* 299 (Lp299 DSM 6595) once daily
[48,49]	↑ Serum albumin concentration compared with control, serum albumin after week 4 compared with baseline, total protein after 4 weeks compared with baseline, and self-assessment of quality of life compared with baseline. ↓ Frequency of vomiting and flatulence compared to baseline. = vomiting and flatulence between groups, total lymphocyte count, body mass, BMI, fat mass content, muscle mass, and total body weight in both groups after 4 weeks of treatment	Two capsules contain 10^10^ UFC of *L. plantarum* 299v (Sanprobi IBS^®^) twice a day
[50]	↓ Antibiotic-associated diarrhea and infectious episodes. = Adverse events probiotic group and the control group.	93 mL of drink (Danactive^®^) with *L. casei* sp. *paracasei* CNCM I1518 (formally DN-114 001) with 10 billion bolused twice daily
[51]	↓ VAP within the intervention group and the incidence of diarrhea. = Time on mechanical ventilation, ICU stay, and hospitalization.	80 mL of a fermented milk product containing 8 × 10^9^ CFU *L. casei* (Shirota strain) (Yakult^®^) administered enterally once daily. The exact amount of *L. casei* was used for oral hygiene once daily
[52]	↓ Duration of fever, incidence of constipation, bactericides, Escherichia coli and Enterococcus, Bacteroides, and serum LPS level. = mortality; length of hospital stay; gastrointestinal adverse effects; and *Bifidobacterium* and *Limosilactobacillus*; content of DAO (intestinal barrier); and IL-10 and TNF-α.	1 tablet of MIYA-BM^®^ with 10^6^ UFC *Clostridium butyricum* 3 times/day
[53]	= Diarrhea cessation; serum albumin; Subjective Global Assessment (SGA) score; food osmolality; and antibiotic use. *B. cereus* was associated with a ↓ diarrhea period (2.5 versus 3.7 days).	4 vials with 5 mL of 10^6^ of *Bacillus cereus* A 05 (Biovicerin^®^) each 6 h or 10 g of soluble fiber (Fiber mais^®^) every 8 h
[54]	↑ Serum levels of total protein, prealbumin, albumin, and transferrin *. ↓ Levels of *Staphylococcus*, *Escherichia coli*, and *Enterococcus*; IgA, IgM, and IgG levels; levels of endotoxin and D-lactic acid; and incidence of gastrointestinal symptoms (compared with the start and with the control)	Probiotic capsules (*Bifidobacterium*) were taken orally, one tablet at a time, tid, or given via tube feeding after being fully dissolved in warm boiled water
[55]	↑ Bowel function. ↓ Duration of ventilation by 40% and length of stay in the ICU by 31%. = inflammatory markers (leukocyte count and CRP levels) in both groups.	*Lactobacillus acidophilus*, *Lacticaseibacillus casei* (former *Lactobacillus casei*), *Lactobacillus lactis*, *Bifidobacterium bifidum*, *Bifidobacterium longum*, and *Bifidobacterium infantis* (30 bilhões de UFC) administered twice daily
[56]	↓ Dietary inflammatory index; h-CRP; serum erythrocyte sedimentation rate level; incidence of diarrhea, abdominal pain, and vomiting compared to control.	300 g/day probiotic (*L. acidophilus* La-5 and *Bifidobacterium lactis* Bb-12) yogurt vs. 300 g/day yogurt
[57]	↓ cases of diarrhea compared with FE; intestinal disorders compared with FF; and length of hospital stay compared with FF. = length of hospital stays between FEB and FE; and total lymphocyte count, albumin, prealbumin, and transferrin levels between groups.	Three groups being fiber-free nutrition formula (FF), fiber-enriched nutrition formula (FE), and fiber- and probiotic-enriched nutrition formula (FEB) (live bifidobacteria and lactobacilli) in tablets
[58]	Time of diarrhea in patient (d = days): T5 = T3 (2 d) > T5 (1.4 d) > T3 (1.2 d, 2 g) > T3 (0.6 d, 3 g > T7 (0.5 d) > T2 (0.33) ↑ (3) diarrhea control (55.8%)	Several groups: (T1) fiber supplement (partially hydrolyzed guar gum and inulin); (T2) partially hydrolyzed guar gum + inulin + *Lactobacillus reuteri* culture) (10 g); (T3) *L. acidophilus*, *L. rhamnosus*, *L. paracasei* and *Bifidobacterium lactis* (1 g, 2 g, and 3 g); (T4) L-glutamine supplement; (T5) synbiotic (1) + probiotic (3) (15 g + 3 g or 10 + 2 g); (T6) synbiotic (1) + L-glutamine (4) (10 + 20 g); (T7) probiotic (3) + L-glutamine (4) (1 + 10 g); (8) fiber (1) + synbiotic of *L. paracasei*, *L. rhamnosus*, *L. acidophilus*, *Bifidobacterium lactis*, and fructooligosaccharides.
[59]	↑ Enteral feed volume, calories, and protein; Intake compared with the first day. = Duration of ENT and days of ENT per day of ICU stay; average energy deficit; enteral feeding intolerance; protein intake; nitrogen balance; and mid-arm circumference measurements.	Symbiotic capsule containing *L. casei* (1.5 × 10^9^ CFU), *L. acidophilus* (1.5 × 10^10^ CFU), *L. rhamnosus* (3.5 × 10^9^ CFU), *Lactobacillus bulgaricus* (2.5 × 10^8^ CFU), *Bifidobacterium breve* (1 × 10^10^ CFU), *Bifidobacterium longum* (5 × 10^8^ CFU), *S. thermophilus* (1.5 × 10^8^ CFU), and fructooligosaccharides
[47]	↓ Gastric residue (57.4% control vs. 30% probiotics); incidence of VAP; length of ICU stays; diarrhea, gastric, and oropharyngeal colonization; and incidence of multidrug-resistant pathogens. = Kaplan–Meier survival curves for time to the first episode of VAP.	2 capsules of probiotic-containing *Limosilactobacillus* (former *Lactobacillus)*, *Bifidobacterium*, and *Streptococcus thermophilus* with 10^10^ CFU
[60]	↑ *Staphylococcus aureus* isolated from the surgical traumas. ↓ Incidence of Surgical Site Infections: Most captured were related to osteosynthesis, followed by facial fractures.	Two sachets of *L. acidophilus* LA-5 (1.75 × 10^9^ CFU), *Lactiplantibacillus plantarum* (former *Lactobacillus plantarum*) UBLP-40 (0.5 × 10^9^ CFU), *Bifidobacterium animalis* subsp. *lactis* BB-12 (1.75 × 10^9^ CFU) and *Saccharomyces boulardii* Unique-28 (1.5 × 10^9^ CFU) twice/day (one through the nasogastric tube and one spread on the oropharynx)
[61]	↓ incidence rate of VAP and sepsis, and length of stay in the ICU compared to the placebo group.	Two sachets of *L. acidophilus* LA-5 (1.75 × 10^9^ CFU), *L. plantarum* UBLP-40 (0.5 × 10^9^ CFU), *Bifidobacterium animalis* subsp. *lactis* BB-12 (1.75 × 10^9^ CFU) and *S. boulardii* Unique-28 (1.5 × 10^9^ CFU) twice/day (one through the nasogastric tube and one spread on the oropharynx)
[62]	↓ Compared with baseline, serum levels of inflammatory factors (IL-6, IL-10, TNF-α, ET-1, and CRP). ↓ ET-1 in 15 days and the concentrations of IL-6, IL-10, and TNF-α in 7 and 15 days; duration of hospitalization and incidence of pulmonary infection; and Glasgow Coma Scale (GCS) in 15 days compared with the control. = 1-month mortality rates, rates of intracranial, incisional, or bloodstream infection, sepsis, septic shock, or systemic inflammatory response syndrome, and Sepsis-related Organ Assessment (SOFA) or Acute Physiology and Chronic Health Evaluation II (APACHE II) scores	6 tablets (210 mg) containing *Bifidobacterium longum*, *Lactobacillus* bulgaricus, and *Enterococcus faecalis* (1.0 × 10^7^ CFU) twice a day by tube or orally
[63]	↑ Overall response rate and albumin and prealbumin levels compared to the placebo group. ↓ Intestinal fatty acid binding protein, diamine oxidase, D-lactate, and 28-day mortality compared to a placebo group.	3 tablets containing *Bifidobacterium longum*, *L. acidophilus*, and *Enterococcus* (Biid-triple Viable Enteric-coated Capsules) three times a day
[64]	↑ stool consistency (BCS value) and Shannon index compared with baseline; increased microbiota biodiversity in half of the intervention patients; propionic and butanoic acids and ketones, such as 2-octanone and 2-pentadecanone, Volatile Metabolome Profile compared with baseline. ↓ “Constipation Scoring System” (CSS) questionnaire compared to the beginning = nutritional measures and hematochemical values; Beta diversity indices and *Lactococcus* spp., *Limosilactobacillus* spp., and *Bifidobacterium* spp. Maintained the same relative abundance compared to the beginning	One sachet Syngut (10^9^ CFU of *L. acidophilus* W22, 3.33 × 10^6^ CFU of *Bifidobacterium lactis* W51, 3,33 × 10^2^ CFU de *L. plantarum* W21, 3.33 × 10^6^ CFU of *Lactococcus lactis* W21, and 0.375 g of Inulin
[18]	= Clinical manifestations of infections; incidence of bacterial infections; CRP levels, intestinal function, and nutritional status between the two groups.	Proxian^®^ probiotic supplement, with *L. plantarum* LP01 (LMG P-21021) ≥ 1 billion live cells/dose, *Lentilactobacillus buchneri* (20 mg) Lb26 (DSM 16341), *Bifidobacterium animalis* subsp. *lactis* BS01 (LMG P-21384) ≥ 1 billion live cells/dose and enriched with zinc (Zn) and selenium (Se)

↑ increase; ↓ decrease; = equal; * Compared with the start and with the control; BMI: body mass index; CRP: C-reactive protein; ET-1: Endothelin 1; hs-CRP: high sensitivity C-reactive protein; ICU: intensive care unit; IL-6: interleukin 6; IL-10: interleukin 10; IgA: immunoglobulin A; IgG: immunoglobulin G; IgM: immunoglobulin M; LPS: lipopolysaccharide; TNF-α: tumor necrosis factor; VAP: ventilator-associated pneumonia.

## Data Availability

Not applicable.
